# Breast Imaging Reporting and Data Systems category 3 (probably benign) breast lesions detected on diagnostic breast ultrasound: The prevalence, outcome and malignancy detection rate in Zaria, Nigeria

**DOI:** 10.4102/sajr.v22i2.1315

**Published:** 2018-11-01

**Authors:** Sefiya A. Olarinoye-Akorede, Garba H. Yunusa, Halima Aliyu, Ahmed U. Hamidu

**Affiliations:** 1 Department of Radiology, Ahmadu Bello University Teaching Hospital, Nigeria; 2 Department of Radiology, Unman Dan-Fodiyo University, Nigeria

## Abstract

**Background:**

Probably benign breast lesions in the Breast Imaging Reporting and Data Systems (BI-RADS 3) constitute a crucial category and a considerable number of all palpable breast masses. Local data concerning the outcome of such lesions in the Nigerian environment is almost non-existent.

**Objectives:**

The goal of this article is to report the frequency, outcome and malignancy detection rate among palpable breast masses that were categorised on ultrasound as BI-RADS category 3 (probably benign) according to the American College of Radiology (ACR).

**Methods:**

Between January 2015 and July 2017, 603 patients had diagnostic whole-breast ultrasound scans. There were 277 women who complained of palpable breast masses, of whom 151 women were diagnosed as having BI-RADS 3 lesions. The final lesion outcome was determined by either biopsy or ultrasound follow-up examination for a total of 2 years. All data were recorded and analysed with Statistical Package for the Social Sciences (SPSS) version 20 (Chicago, USA).

**Results:**

The frequency of BI-RADS category 3 lesions among all the women who underwent breast ultrasound was 25% (151/603); and 54% (151/277) in patients with palpable breast masses. There were 25 patients who were excluded because of incomplete data or who were lost to follow-up. A total of 122 patients had both ultrasound examination and histopathologic diagnosis, while only 4 were followed up for 2 years on ultrasound alone. Of the 122 women biopsied, 117 (95.9%) had benign histologic outcomes, and of the remaining 5, cancer was confirmed in 2 (1.6%), while the remaining 3 patients (2.5%) had lesions considered intermediate at histology (juvenile papillomatosis, borderline phylloides and atypical ductal hyperplasia). Three out of four patients who had ultrasound follow-up alone had stable lesions after 2 years, while one patient had complete resolution.

**Conclusion:**

This study found a significantly high biopsy rate of 80% (122/151) for probably benign lesions but a low detection rate for malignancy (1.6%). Follow-up with imaging rather than biopsy for lesions sonographically described as probably benign, will reduce medical costs and unwarranted invasive procedures.

## Introduction

The ‘probably benign’ assessment (category 3) in the Breast Imaging Reporting and Data Systems (BI-RADS) is assigned to lesions with specific imaging findings. These include a solid mass with an oval shape, circumscribed margin, parallel orientation, homogenous echo texture and no suspicious malignant characteristics.^1,2^ While these masses have benign imaging features, there is still a low (< 2%) risk for malignancy.^3^ Radiologists have a key role to play in deciding on the definitive management as to whether the mass should be biopsied or not. The American College of Radiology (ACR) management recommendation is a short-interval imaging follow-up usually at 6, 12 and 24 months, which is considered a reasonable alternative to biopsy.^1,4,5,6^

For the purposes of auditing, a BI-RADS 3 assessment has been reclassified recently by the 5th edition of BI-RADS as audit positive (previously audit negative) for all screening modalities – mammography, ultrasound and MRI.^7^ In contrast, for diagnostic imaging, BI-RADS 3 assessment is considered as audit negative.^7^ Nonetheless, there is a general low threshold for biopsy even when the imaging features are benign.^8^ This situation is also observed in Nigeria.

In this study, we report on the frequency of probably benign breast lesions, the outcome and the detection rate for malignancy in Zaria, with the purpose of reviewing the biopsy-driven practice for masses with benign features.

## Materials and methods

The ethics committee of Ahmadu Bello University Teaching Hospital approved this retrospective review of 603 patients who had whole-breast diagnostic ultrasound examinations between January 2015 and July 2017 (ABUTH/HREC/D38/2018). Of these, 151 women had palpable breast masses that were classified as probably benign (BI-RADS 3). We excluded 25 patients with incomplete data or loss to follow-up and finally recruited 126 patients for this study (122 had both ultrasound and biopsy; 4 had ultrasound follow-up alone at 6, 12 and finally 24 months).

High-resolution ultrasound scans of the breasts were performed by the breast radiologists using a Mindray ultrasound machine (DC-8, China), equipped with a 7 MHz–12 MHz linear transducer. Physical examination of the breast and axilla was performed in the seated position, noting relevant findings before commencing the scan. With the arms raised, each breast was firstly scanned in the supine position for the medial portions of the breast, then in the contralateral posterior oblique position for the lateral breast portion.

Overlapping transverse and longitudinal ultrasound scanning technique, as well as radial and anti-radial scanning was done in real time until the entire breast was scanned. The nipple–areolar complex and lastly the axilla were also examined with ultrasound.

The images were evaluated in a team context by the performing breast radiologists (with 4 and 9 years of experience) using the ACR BI-RADS 4th edition (Ultrasound 1st edition),^2^ the Stavros criteria,^3^ as well as personal experiences in our institution. The cases selected were those that satisfied the criteria for BI-RADS category 3, namely a solid mass with an oval or gently lobulated shape, circumscribed margin, uniform echotexture and a parallel orientation. Complicated cysts were also classified as probably benign. Lesions that were definitely benign like simple cysts and normal lymph nodes were excluded, as were lesions with any suspicious features like irregular margins, antiparallel orientation or posterior shadowing.

The standard of reference for lesion outcome was histopathology or 2 year follow-up (at 6 months, 1 year and 2 years). All data were recorded and analysed using SPSS version 20 (SPSS Inc., Chicago, IL, USA). Figures and tables were used to show descriptive frequency distributions.

## Results

The mean age of the patients was 29.7 ± 11.3 years (range 13–68 years). The modal age of the patients was 20–29 years, accounting for 47% (Table 1).

**TABLE 1:** Age distribution of patients with breast masses diagnosed as probably benign.

Age (years)	Frequency	%
<19	21	13.9
20–29	71	47.0
30–39	28	18.5
40–49	19	12.6
50–59	10	6.6
60–69	2	1.3

**Total**	**151**	**100.0**

The frequency of probably benign lesions from this study was 151/603 (25.0%). It also constituted 54%, or 151 of the 277 women with palpable lesions. Outcome data were available for 126 patients, which was either histology (*n* = 122) or ultrasound follow-up after initial ultrasound examination at 6, 12 and 24 months (*n* = 4) (Table 2).

**TABLE 2:** Outcome of Breast Imaging Reporting and Data Systems 3 lesions.

Lesion outcome	Frequency of biopsy findings	
	*n*	%
**Patients sampled for biopsied (*n* = 122)**		
Cancers detected	2	1.6
Benign histology	117	95.9
Intermediate	3	2.5
**Patients followed with ultrasound (*n* = 4)**		
Cancers detected	0	0.0
Resolved and downgraded to BI-RADS 1	1	25.0
Stable and downgraded to BI-RADS 2	3	75.0

BI-RADS, Breast Imaging Reporting and Data Systems.

**FIGURE 1:**
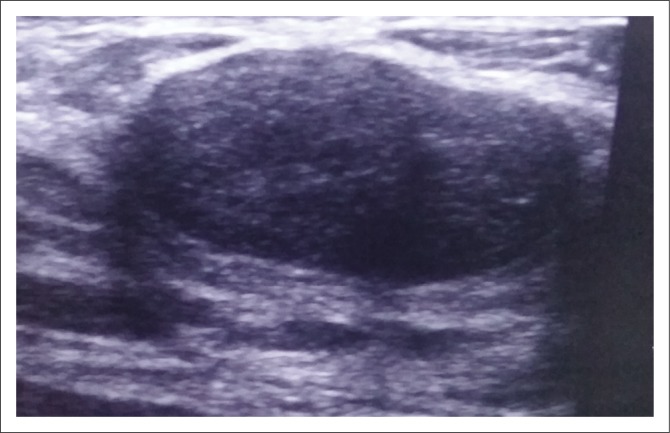
Palpable right breast lump in an 18-year-old woman noticed 6 months previously. Ultrasound showed an oval-shaped homogenous hypoechoic mass with well-defined margins. Posterior acoustic enhancement and edge shadowing are seen. Histology confirmed the diagnosis of a fibroadenoma.

**FIGURE 2:**
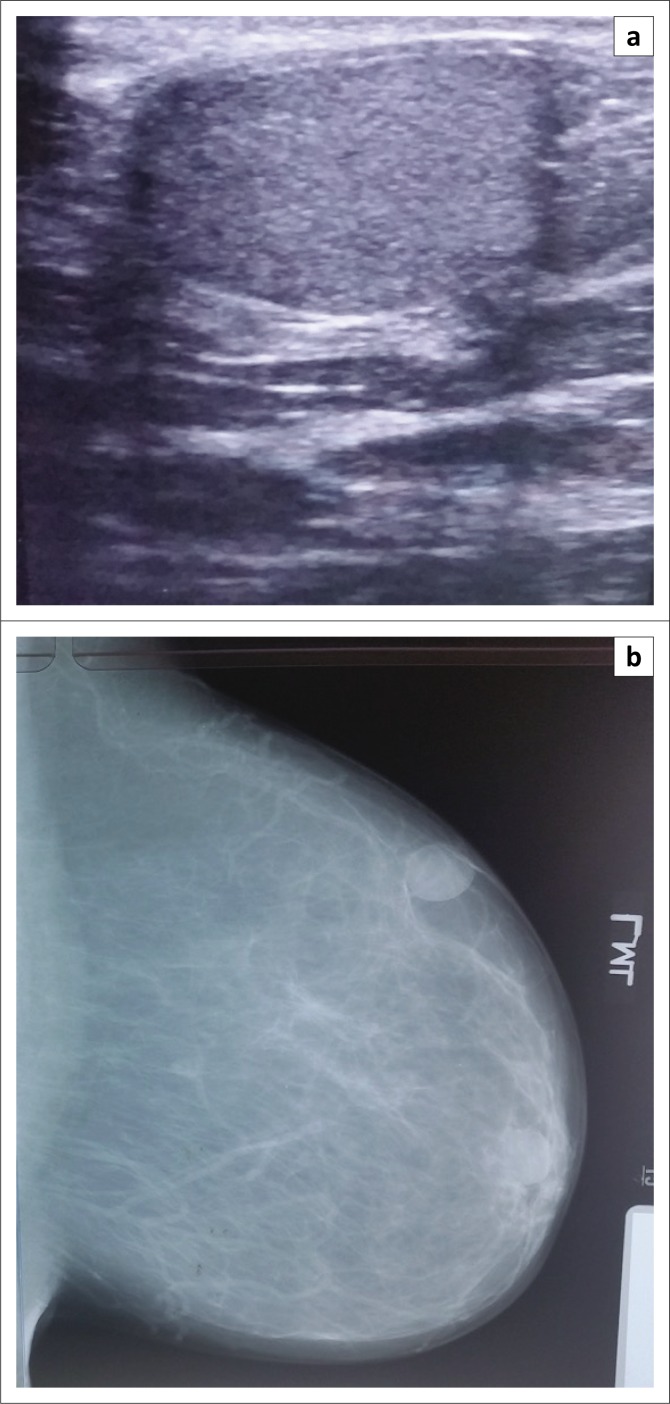
A palpable left breast lump in a 48-year-old woman. She had a breast trauma 3 years prior to presentation: (a) Ultrasound of the left breast showed a well-defined hyperechoic mass and (b) left medio-lateral oblique mammogram showing a mass with a calcified (egg shell) rim. At the patient’s request, she had a biopsy, which confirmed an oil cyst (from post-traumatic fat necrosis).

Of the 122 patients with histologic confirmation, 117 (95.9%) had a benign outcome; out of these, 74 (63.24%) were fibroadenomas (Figure 1). Others, shown in Table 3, included fibrocystic disease, lactating adenoma, benign ductal papilloma, duct ectasia, chronic inflammatory mass, oil cyst (Figures 2a & 2b) and galactocoele.

**TABLE 3:** Types of benign breast lesions.

Lesion	*n*	Frequency (%)
Fibroadenoma	74	63.24
Fibrocystic disease	12	10.25
Ductal ectasia	7	5.98
Abscess or inflammatory	6	5.10
Clustered microcysts	3	2.56
Lactating adenoma	3	2.56
Galactocoele	3	2.56
Fat necrosis	2	1.70
Fibroadenomatoid hyperplasia	3	2.56
Benign ductal papilloma	4	3.41

**FIGURE 3:**
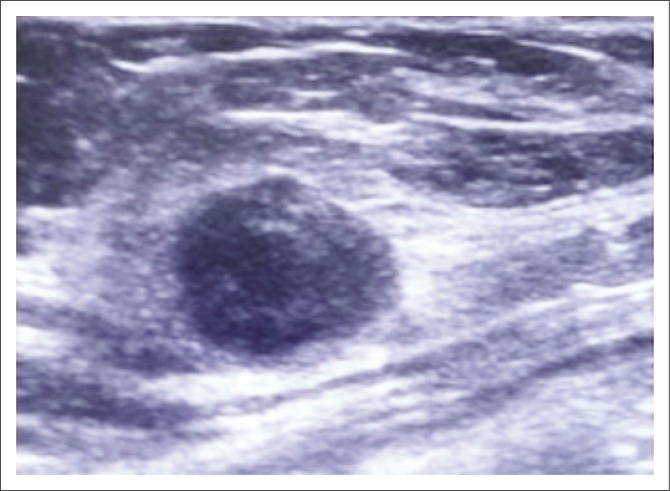
Left breast lump in a 44-year-old woman 3 years prior to presentation. Ultrasound examination showed a round hypoechoic mass with circumscribed margins and posterior acoustic enhancement. Ultrasound assessment was Breast Imaging Reporting and Data Systems 3 (probably benign). Histologic diagnosis was invasive ductal carcinoma.

Of the remaining five women, three had intermediate (borderline) lesions, which included juvenile papillomatosis, borderline phylloides and atypical ductal hyperplasia. Invasive ductal carcinoma (Figure 3) was detected in the other two women. Overall, the malignancy detection rate from this study was therefore 1.3% (2/151).

The patients who were followed up with ultrasound alone had stable lesions after 2 years (*n* = 3) with no symptoms and were downgraded to BI-RADS 2 (benign). These were fibroadenomas (*n* = 2) and ductal ectasia (*n* = 1). Complete resolution occurred in a patient with Mondor’s disease, which was downgraded to BI-RADS 1 (negative).

## Discussion

Probably benign masses are a frequent finding in ultrasound examinations of the breast. It constitutes a problematic category because its definitive management is dependent on whether it is upgraded to malignancy or downgraded to benign. In this study, it accounted for 25% of all breast ultrasound examinations. This is similar to the reports of 25% by Barr et al.^9^ in the USA and 24.9% by Moon et al.^10^ in Korea. This study constituted mostly younger women between 20 and 29 years old (mean age 29.7 ± 11.3; range of 13–68 years). In other studies, the women were older as reported by Moon et al. (mean 44.5; range 15–78 years);^10^ Park et al. (mean 34, range 12–64 years)^8^ and Harvey et al. (mean 44.4; range 12.2–87.8 years).^11^

The ACR recommendation for management of BI-RADS 3 lesion is short-term ultrasound follow-up at 6, 12 and 24 months.^2,7^ Although very few (four) of our patients were compliant with this recommendation, which spanned 2 years, the lesions that we evaluated with only ultrasound follow-up had benign outcomes as with previous findings of Alimoglu et al. Moon et al. and Raza et al.^1,10,12^ The authors believe that one of the reasons for poor compliance could be the lack of evidence in the community to support low prevalence of malignancy for probably benign breast lesions; hence the high biopsy rate. This study aimed to address this. Also, anxiety from fear of cancer could make patients opt for biopsy, which is quicker, as observed by Harvey et al.^11^ Three of our patients had stable lesions after 2 years, while in one patient the mass completely resolved. In the study by Alimoglu et al.^1^ 70% of the lesions remained stable during follow-up, 13.1% showed interval regression while 14.0% showed interval progression. They noted that the majority of the interval changes occurred within the first 2 years. In contrast, in the study by Moon et al. 906 out of 920 (98.5%) turned out benign.^10^

The rest of our patients (122 of 126) had tissue sampling. Of these masses, 95% were histologically benign. The commonest histologic finding was fibroadenoma. This was not surprising given that fibroadenoma is the commonest mass in young African women. Fibrocystic disease is mostly seen in the fourth decade and malignancy in the fifth decade.^13^

The malignancy rate of 1.3% found in this study correlates with the cancer detection rate of less than 2% that is associated with breast masses that are categorised as probably benign.^6,12,14,15^ Lower cancer detection rates than ours (0.2%, 0.7%, 0.8%) among BI-RADS 3 lesions have been previously reported by Graf et al. Chae et al. and Baum et al.^6,16,17^ However, in these studies, ultrasound was combined with mammography, which improved diagnostic accuracy. MRI BI-RADS category 3 is not frequently used. It is associated with higher malignancy detection rates when compared with ultrasound or mammography.^18^ This is likely because the criteria for MRI BI-RADS category 3 are not well established as it is a relatively new modality.

Our study replicated the fact that the risk for breast cancer is less than 2% for BI-RADS 3 lesions. When this is considered against the background of significantly higher cost of biopsy in our centre, imaging short-term follow-up would be ideal. Also, unnecessary high tissue sampling of benign masses would be avoided. Initial 6 month ultrasound follow-up will be critical to detect rapidly growing masses because we noticed that our patients’ compliance declined with long follow-up intervals.

However, where the patient is not able to return for a follow-up examination or if imaging is not available to the patient, they should be offered an immediate biopsy.^19^

## Conclusion

Ultrasound is a cost-effective alternative to biopsy for benign breast lesions. However, patients should be carefully assessed before their lesions are assigned to the BI-RADS 3 category. Physicians and radiologists need to explain the goals of follow-up with their patients effectively to ensure better compliance and reduce anxiety.

## Limitations

There were 25 patients who were excluded for loss to follow-up and incomplete data, thus creating an unavoidable bias. The small sample size limits interpretation. Nonetheless, our results are in accordance with previous studies that involved larger sample sizes.
